# Gene–Environment Interplay Between Physical Exercise and Fitness and Depression Symptomatology

**DOI:** 10.1007/s10519-020-10009-9

**Published:** 2020-08-14

**Authors:** Wendy Johnson, Erik Lykke Mortensen, Kirsten Ohm Kyvik

**Affiliations:** 1 Department of Psychology, University of Edinburgh, 7 George Square, Edinburgh, EH8 9JZ UK; 2 Department of Public Health, University of Copenhagen, Copenhagen, Denmark; 3 Department of Epidemiology, University of Southern Denmark, Odense, Denmark

**Keywords:** Depression, Gene–environment interplay, Physical fitness, Physical exercise, Neuroticism

## Abstract

Studies often report beneficial effects of physical exercise on depression symptomatology, both in clinical and community samples. In clinical samples, effects are observed using physical exercise as primary treatment and supplement to antidepressant medications and/or psychotherapies. Magnitudes vary with sample characteristics, exercise measure, and study rigor. Both propensity to exercise and vulnerability to depression show genetic influences, suggesting gene–environment interplay. We investigated this in a Danish Twin Registry-based community sample who completed a cycle fitness test and detailed assessments of depression symptomatology and regular exercise engagement that enabled estimates of typical total, intentional exercise-specific, and other metabolic equivalent (MET) expenditures. All exercise-related measures correlated negatively with depression symptomatology (− .07 to − .19). Genetic variance was lower at higher levels of cycle fitness, with genetic and shared environmental correlations of −  .50 and 1.0, respectively. Nonshared environmental variance in depression was lower at higher levels of total MET, with no indications of genetic or environmental covariance. Being physically active and/or fit tended to prevent depression, apparently because fewer participants with higher levels of activity and fitness reported high depression symptomatology. This was driven by nonshared environmental influences on activity but genetic influences on physical fitness. Genetic correlation suggested people less genetically inclined toward physical fitness may also be genetically vulnerable to depression, possibly because inertia impedes activity but also possibly due to social pressures to be fit. Exercise programs for general well-being should emphasize participation, not performance level or fitness. We discuss possible interrelations between fitness aptitude and metabolism.

## Introduction

The World Health Organization (WHO; Depression Fact Sheet, March 2018) considers depression the leading cause of worldwide disability, with currently more than 300 million sufferers at any time, and rising prevalence rates. Even without reaching the severe levels seen in clinical patients and pathological depressions, depression has huge personal and economic costs. Beyond distress, it disrupts work and school performance and social and family relationships, contributes to many physical health impairments, and sometimes leads to suicide. This makes identifying both factors contributing to its development and effective means of preventing and treating it crucial.

Physical activity has been identified as one of reducing and/or preventing the symptoms, suffering, and consequences of depression, but it appears, like most treatment approaches, to be more effective for some people than others (e.g. Choi 2019; Krogh et al. 2017; Perez-Lopez et al. 2017; Weinstein et al. 2017). It is not known why this is the case, but about half those experiencing depression do not have access to formal treatments. In many countries treatment is received by as few as 10% of those needing it (WHO 2018) due to poor access, limited available trained providers, stigma associated with mental health impairment, and/or inaccurate assessment. This, coupled with minimal associated costs and potential risks, make understanding when, to what degree, and in whom physical activity has positive effects on depression.

Physical activity also has the advantages of being of known benefit at least to physical health. Evidence of effectiveness in reducing depression symptomatology has been strong enough that exercise has been adopted as a general recommendation by organizations such as the United Kingdom’s and Canada’s National Health Services, and the Mayo Clinic. Moreover, many people who have been depressed and gotten involved in exercise programs proclaim that exercise has been critical to recovering their mental health (e.g., browse any issue of *Runner*’*s World*). This is at best anecdotal evidence of course, but the placebo effect is powerful.

Among possible reasons for inconsistent study indications of effectiveness of exercise are that both propensity to exercise and depression show genetic influence (e.g., Petterson et al. 2019; Zhang and Speakman 2019), and one of the primary symptoms of depression is lack of motivation to do much of anything. This combination, coupled with other common depressive symptoms such as sleep disruption, chronic lack of energy, helplessness, and low self-esteem, can make even contemplating exercise rather daunting. This means that those most genetically vulnerable to experiencing depression could also often be least genetically able to experience exercise’s potential to remediate it, if only because they are more likely to experience the kinds of symptoms that make getting going with it difficult. Few studies have examined this, but two that did (De Moor et al. 2008; Stubbe et al. 2007) were consistent with it. That is, though in unrelated pairs of people, exercisers tended to have higher life satisfaction and lower depression and anxiety, these associations were reduced in dizygotic twin pairs and non-existent in monozygotic twin pairs.

In situations such as this, where evidence of causal linkage is present but inconsistent, there are genetic influences on both purported cause and outcome, and the genetically influenced characteristics associated with purported cause are more likely to involve behavioral choices, it has been quite consistently observed that population-level variance in outcome was constrained at higher levels (doses) of purported cause. Moreover, it has often been the genetic variance, rather than variance attributable to between- (shared) or within-family (nonshared) environmental influences, that has been constrained, though this has not always been the case. For example, perhaps with particular relevance, Johnson et al. (2015) observed that variance in most of six different measures of body adiposity was lower at higher levels of both typical metabolic-equivalent energy expenditure of task (MET) and cycle fitness, as have several other studies (e.g., Ahmad et al. 2013; Heitmann et al. 1997; Karnehad et al. 2006; Kilpelainen et al. 2011, 2009; Rankinen and Bouchard 2012; Silventoinen et al. 2009; Williams 2011). Importantly, however, (Johnson et al. 2011) extended prior work to examine particular variance components that were moderated differed among the adiposity measures in ways offering indications of the involved social, psychological, and metabolic pathways that could be helpful in designing interventions using exercise to combat obesity with further confirmation and test. Other examples include various measures of socioeconomic status and a wide range of health outcomes (e.g., Dinescu et al. 2016; Hicks et al. 2009; Johnson and Krueger 2005a, b).

The purposes of this study were thus to investigate the possibility that exercise constrains variance in depression symptoms too and to explore what could be learned from the genetic and environmental pathways involved that could be used to maximize the benefits to be realized and the proportions of the population that could experience them. To do this, we made use of a community-based twin sample representative of healthy adults in the Danish population who had been assessed using three well-known measures of depression symptoms and two measures of typical physical activity levels—one an objective measure of aerobic physical fitness and the other a self-report of typical engagement in a large variety of kinds of physical activity.

Of course, as noted above, it is highly possible, even likely, that, to whatever extent associations between exercise and depression levels prevail, they are reciprocal in nature. Not only may exercise limit, prevent, or remediate depression symptomatology, but depression symptoms may directly limit willingness/ability to engage in exercise. We thus fit models using both potential directions of moderation.

## Methods

### Participants

Our study participants were surveyed on a much larger range of physical and psychological variables than used in this study, and have been featured in many other papers examining those other variables. They participated in the GEMINAKAR study, which was designed to explore how genetic and environmental influences are involved in associations between lifestyle factors such as smoking, diet, and exercise and endophenotypes of the metabolic syndrome, a precursor of many chronic illnesses such as diabetes, heart disease, stroke, and vascular dementia. The study was given ethical approval by the Danish Data Protection Agency and all the Danish Regional Ethics Committees and conducted in compliance with the Helsinki Declaration. Participants were recruited from the nationwide (Skytte et al. 2013), established in 1954, via letter from 1997 to 2000, giving informed consent for GEMINAKAR when they arrived for assessment. GEMINAKAR made use of 756 complete twin pairs [783 women, 729 men; 311 monozygotic (MZ), 314 same-sex dizygotic (DZ), 131 opposite-sex DZ] then aged 18–67 (median 38.0) who did not abuse alcohol or drugs, were not pregnant or breastfeeding, had not been diagnosed with diabetes or heart disease, and did not have any other physical condition that would have precluded participation in the aerobic physical fitness test (Benyamin et al. 2007; Hasselbalch et al. 2008; Schoesboe et al. 2004). The assessment included detailed physical examinations and blood sampling following a 12-h fast, with a light meal before the aerobic fitness test. All assessments were conducted in one of two identically equipped locations in Copenhagen and Odense by trained medical examiners. Zygosity was determined using DNA-base microsatellite markers from the AMPFISTR Profiler Plus Kit made by PE Applied Biosystems, Perkin Elmer, located in Foster City, CA, USA.

### Measures

#### Physical Activity

For the test of aerobic physical fitness, participants pedaled a stationary bicycle mounted with an ergometer quantifying energy expenditure. They were instructed to start at a workload of 35 W and to increase it every 2 min by 35 W until they could sustain no more. The highest workload reached, the seconds over which it was maintained, age, sex, and weight were used to assess VO_2max_ in l/(kg min). VO_2max_ is a measure of maximal oxygen consumption during exercise of increasing intensity, and considered an indication of cardiorespiratory fitness and physical endurance capacity. These, in turn, reflect both inherent capacity and extent of exercise exposure or training, to different degrees in different people. That is, there are clear individual differences in inherent biological factors related to muscular strength and oxygen transport that are independent of physical activity (Joyner and Lundby 2018), and people appear to differ considerably in VO_2max_ response to exercise training, in extent of response, training intensity necessary to generate it, and time frame over which it occurs. Both VO_2max_ level and its response to training also vary with age and sex and show considerable genetic influence (e.g., Bouchard et al. 1999; Klissouras 1973), though the specific genes involved are not yet clear (Williams et al. 2017). Thus, though it was objective, this bicycle test only indirectly assessed current typical physical activity.

MET was assessed based on an extensive series of questions about participants’ current typical patterns of physical activity. They reported the extents to which their jobs involved sitting, standing, walking, lifting, and heavy manual labor, and whether any lifting was light, moderate, or heavy. They rated their leisure-time physical activity similarly, and reported the typical number of hours they walked or cycled per day in summer and winter in half-hour increments to 2 h, 1–2 h, and 2+ h, and rated the speed at which they did so as ‘slow’, ‘normal’, ‘brisk’, or ‘very brisk’. As well, they reported how many minutes per week they engaged in sports such as running, tennis, swimming, ‘fitness studio’, football/soccer, handball, gymnastics, etc. Using the compendium of typical MET expenditures compiled by Ainsworth et al. (2011), we used all this information to estimate average total daily MET expenditure, MET expenditure for activities often intended explicitly at least partly as exercise (walking, cycling, and sports), and MET expenditure the rest of the time for each participant. Measured in kg/kcal/h, METs reflect metabolic expenditure as ml/kg/min oxygen uptake, thus also reflecting VO_2max_. Thus, in addition to being based on subjective reports of engagement in exercise, total daily METs reflect inherent individual biological factors varying with age and sex and subject to genetic influences that are related to overall bodily function and response to exercise as well as engagement in activities intended as exercise. We thus focused particular interest on METs for exercise but estimated all our models separately for total daily METs, METs for exercise, and Other METs so that we could attempt to distinguish pathways reflecting inherent biological factors related to overall bodily function from those related specifically to engagement in intended exercise. But published MET values for specific activities are overall averages of ratios of MET expenditure during activity to that at rest, derived under specific experimental conditions from specific samples. Actual individual levels of expenditure depend on intensity of effort, fitness, and amount of mass in motion, and can vary even in the same person from those in experimental conditions. Thus our estimates of METs for exercise were subject to their own specific additional limitations. Despite all this, our measures were considerably more extensive than those used in most studies, which have often been based on single questions with 4–5 response options (e.g.).

#### Depression

Participants completed the 240-item NEO-PI-R (Costa and McCrae 1992), which includes an 8-item measure of depression among its facets. They also completed the 13-item Symptom Checklist Depression scale (Hanson et al. 2014) and the 40-item Obvious Depression Scale of the Minnesota Multiphasic Personality Inventory (Burkhart et al. 1980). All have been well-validated as measures of depression. We standardized each of them and used the mean of the standardized values as a composite measure of depression symptomatology. We regressed age, age-squared, sex, and their interactions from all five variables prior to analysis.

#### Analysis

That variance in individuals’ characteristics can be decomposed into components separably attributable to additive genetic (A) and shared (C) and nonshared (E) influences is the foundation of the quantitative genetic twin model. Simply put, greater similarity (higher correlation) between MZ than DZ twins indicates genetic influence because MZs share effectively all their genes, but DZ only on average 50% of their segregating genes. DZ correlation greater than half the MZ correlation indicates shared environmental influence (within but not between twin pairs so that they make the former similar but distinguish among the latter), while MZ correlation less than 1 indicates nonshared environmental influence (measurement error and anything that acts differently on family members). This model, and to varying degrees its extensions, requires some rather strong assumptions, some of them pretty much ubiquitously violated. These include that twins are ‘just ordinary folks’ (Johnson et al. 2002), generally quite accurate in the sense that twins represent the general population of primarily singletons; there is no assortative mating for the characteristic in question (violated badly for some but not all characteristics); that MZ and DZ twins do not experience environmental influences systematically differently by twin type; and that genetic and both kinds of environmental influences act completely independently of each other—do not correlate or interact in any way (importantly, basically ubiquitously badly violated). We know exactly what kinds of effects violations of these assumptions have on model results, but we generally do not know which or to what degrees violations are present (Johnson 2007; Purcell 2002). This was the case in this study as well, but, as described below, the models we used relaxed some of these assumptions.

**Fig. 1:**
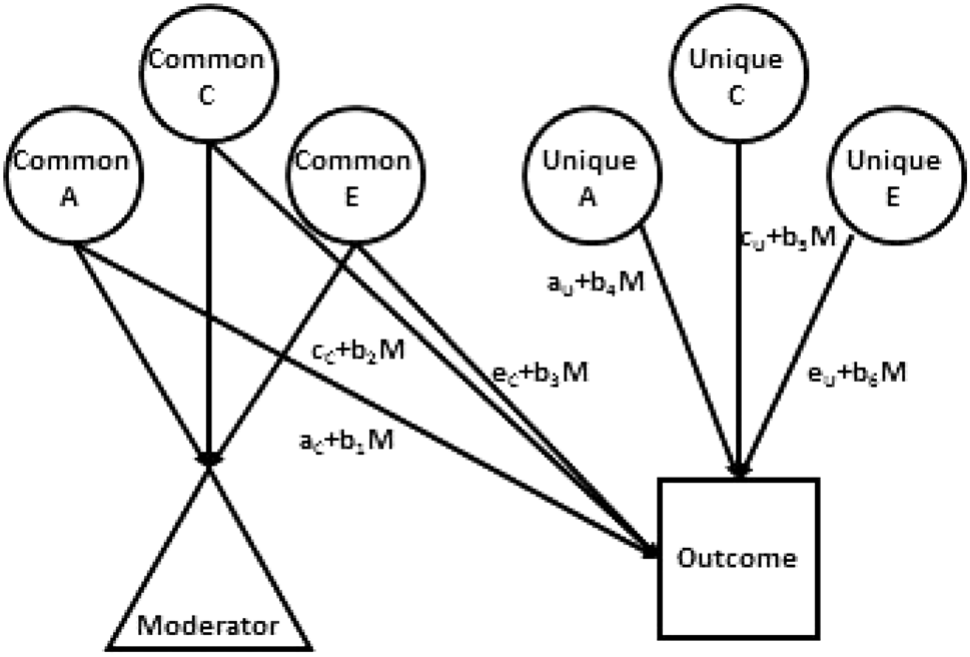
The full gene–environment correlation model used as primary to test gene–environment moderating effects. *A* genetic, *C* shared environmental, *E* nonshared environmental influences, *M* moderating variable

An important extension to this univariate model is simultaneously to estimate the A, C, and E influences on at least two characteristics. This model, known as a ‘Cholesky model, produces separate A, C, and E estimates of the variances unique to each trait and those of the covariances between them. We ran it for each of our variable pairs to serve as base information with which to compare our variance-moderation models. Our primary moderation model provided this information too, but in addition relaxed the assumption that genetic and both kinds of environmental influences are independent by allowing one variable’s variance components to moderate those of the other variable so that that variable’s variance components differed systematically among people with different levels of the moderating variable (Johnson 2007; Purcell 2002). Figure 1 diagrams this model. The variance-moderating parameters are the b’s, which pertain to A, C, and E components common to the moderator and ‘outcome’ variable and unique to the outcome variable. The model is thus based on assumption that the moderator acts causally on the ‘outcome’ variable. Because exercise could affect depression but depression could also affect exercise levels, we estimated all models in both directions to reflect the likelihood of reciprocal influences between the two variables.

This model estimates only variance components; it does not estimate direct main mean effects that apply uniformly to everyone or even track how mean levels of the outcome variable vary with levels of the moderating variable. Thus, it is possible that it inappropriately indicates moderated covariance when the actual situation is direct nonlinear main effects. To address this, we also ran a model estimating moderation only on variance unique to the outcome variable and nonlinear main effects in lieu of moderating covariance effects (Rathouz et al. 2008) and considered the extended main effects model proposed by van der Sluis et al. (2012) where relevant.

We implemented all models in Mx (Neale et al. 2007) software using maximum likelihood estimation so that all data were included, regardless of availability of co-twin data. Our primary interest was absolute levels of variance so we present those rather than the more common proportions of total variance. Scale of measurement that produces non-normally distributed variables can produce artefactual variance moderating effects. This is especially true of the scales on which all our individual depression measures, and thus our composite, are based. They have no clear relations to actual depression severity, so it would not be unreasonable to transform our composite in particular purely for methodological reasons. To evaluate this, we checked that all variables were approximately normally distributed and that equal intervals along their ranges showed no variance trends (Falconer and McKay 1989), which minimizes the possibly that model-indicated moderation could result solely from variables’ distribution properties. Even after age–sex adjustment, the depression composite was somewhat skewed (1.16). We thus log-transformed it and ran all models using both versions. Results were similar, further supporting inference that model-indicated moderation was not statistical artifact. We thus present only those using the untransformed variable to preserve the intended measurement scale and what was probably a legitimate observation about this relatively healthy community-based sample: that severe depression was rather rare.

We worked to produce the most parsimonious model for each variable pair, trying first to drop the covariance moderating (or quadratic main effect in those models) parameters without significantly reducing model fit. We did this because, when it was possible, it eliminated the problem of confounded main mean-level effects and moderated covariance. Beyond that, however, we handled each modelled set of variables individually, first replacing any needed covariance moderators (or quadratic main effects). There was no need for a formulaic approach as it was immediately clear in the full models when individual moderating (or nonlinear main effect in those models) parameters could be dropped without losing model fit due to the magnitudes of their estimates. We this dropped those all at once and then any systematically tested all possible remaining unique variance moderating parameter combinations. We dropped parameters not to eliminate the possibility of their existence but to focus on the most important pathways in this largely exploratory study. We compared models using the chi-square difference test, and the information-theory fit statistics Akaike Information Criterion (AIC; Akaike 1983) and the Bayesian Information Criterion (BIC; Raftery 1995), and Sample-Size Adjusted BIC (SSABIC). For all these fit statistics, lower values indicate better model fit, but do not offer indications of absolute goodness of fit, The chi-square difference test is the least robust because it is the most heavily dependent on sample size and does not reflect model parsimony. The others all do—to accomplish that was the reason for their development—but they do so to varying extents in varying circumstances. BIC is the most sensitive to model parsimony and tends to be overly sensitive in larger samples, hence the development of SSABIC. For our kinds of sample and models, AIC and SSABIC tend to perform best (Markon and Krueger 2004), and we relied most on them in judging which models fit best when fit indications were not consistent. In a couple cases, it would have been reasonable to select the model indicating no moderation, but we did not. We selected as we did because this was not the only reasonable selection and there were substantive reasons to suspect that there was moderation we lacked power to detect clearly and we believed it was important to try to obtain as complete a picture of the gene–environment interplay as possible. We focused on indications of parameter significance based on the model-fit criteria rather than confidence intervals in interpreting models, as the outcome variable variance component estimates could vary with moderator and the estimates of genetic and environmental correlations were calculated based on the parameter estimates rather than directly estimated themselves. Even in basic Cholesky models, the genetic and especially the shared environmental correlations can rarely be estimated very precisely because they compound error in the variance component estimates of both involved variables with violations of the assumptions underlying the models, so the estimates we present need to be considered suggestive.

## Results with Intermittent Discussion

Table 1 shows the raw descriptive statistics for all variables. As expected for a healthy community sample, depression levels as indicated by all three scales tended to be relatively low, but the full ranges of scores were quite well represented. Cycle Fitness and MET expenditures for the various kinds of exercise varied widely, much more so than did Other MET. This too would be expected because everyone expends some base metabolic energy in sleeping, eating, simply sitting around, and even breathing, but not everyone exercises at all, and some who do train very athletically and/or engage in particularly energetic forms of recreation, while others exercise primarily via cycling for transportation, daily walks, or other relatively leisurely activities, Consistent with this, Total MET for Exercise indicated that quite a few participants got none while others were likely actively athletic.

**Table 1:** Raw descriptive statistics

All	N	Minimum	Maximum	Mean	Deviation
Age	1512	18	67	37.8	10.9
NEO-PI-R Depression	1213	0	32	12.6	5.2
SCL-Depression	937	0.00	2.38	0.295	0.347
MMPI Obvious Depression	1179	1	32	9.0	4.3
Cycle Fitness Test Rating	1248	14.7	57.7	34.34	8.10
Daily Total MET Expenditure	1244	1475	6000	3283.1	928.9
Daily MET Expenditure in Sport	1512	0	1800	104.4	170.2
Daily MET Expenditure Walking	1512	0	825	208.1	177.5
Daily MET Expenditure Cycling	1512	0	1050	83.4	113.4
Daily MET for Exercise	1512	0	2220	395.9	321.5
Other Daily MET Expenditure	1244	1440	5130	2801.9	772.2

Correlations among the raw study variables are shown in Table 2. Depression scores varied very little with age, but as would be expected, physical activity levels decreased with age noticeably (except for walking), especially Cycle Fitness, the objective measure. Even Other MET decreased slightly, probably indicating general metabolic slowing. As is typical, women tended to display more symptoms of depression, and they tended to have lower Cycle Fitness. Their Total MET expenditure tended to be lower, but this was because Other MET tended to be lower, as they tended to expend more MET on all the exercise activities, especially Walking but also Cycling. Their lower Other MET probably primarily reflected their generally smaller body size. The depression scales correlated well, all about .6, justifying our treating them as a single composite variable.^1^ All the physical activity measures were negatively correlated with all the depression scales, generally in the range of −  .1 to −  .2. Even Other MET was negatively correlated with the depression scales to similar degrees, with the indicated lower general metabolic rate potentially indicating either depression symptoms or vulnerability to them—or both. Cycle Fitness was moderately (.3+) correlated with Total, Sport, Cycle, and Exercise MET, but not even significantly with Walk MET, indicating that walking, at least as often used as exercise, may do little to build cardiorespiratory fitness unless it is extremely low. To whatever extent this is true, walking could still remediate depression symptoms by getting people outside in fresh air and affording access to green spaces, social engagement, change of scenery, and relief of muscle cramping and tension from long periods in seated positions. Cycle Fitness was also correlated (.25) with Other MET, potentially indicating that exercise boosted metabolic levels or that people with higher general metabolic levels were more likely to exercise—or both. All the individual activity METs correlated about .2: people who were active in one area tended at least somewhat to engage in others too. Table 3 shows the correlations among the variables actually used for analysis, after forming the Depression and MET for Exercise composites and regressing age and sex from them. They were very similar to those for the raw variables, indicating little reason to consider either age or sex as likely important moderators.

**Table 2:** Correlations among Raw Study Variables

	1	2	3	4	5	6	7	8	9	10	11	12
(1) Age	1.000											
(2) Female Sex	.053	1.000										
(3) NEO Dep	− .043	.179	1.000									
(4) SCL-Dep	− .061	.162	.630	1.000								
(5) MMPI Dep	.019	.148	.641	.591	1.000							
(6) Cycle Fitness	− .460	− .312	− .153	− .141	− .240	1.000						
(7) Total MET	− .139	− .179	− .081	− .137	− .127	.315	1.000					
(8) Sport MET	− .135	.097	− .104	− .130	− .135	.331	.465	1.000				
(9) Walk MET	.033	.288	− .040	− .073	− .035	.044	.480	.204	1.000			
(10) Cycle MET	− .161	.227	− .006	− .040	− .032	.303	.227	.197	.252	1.000		
(11) Exercise MET	− .110	.290	− .089	− .137	− .116	.341	.650	.711	.749	.596	1.000	
(12) Other MET	− .100	− .181	− .064	− .113	− .109	.245	.958	.296	.337	.077	.405	1.000

Correlations in excess of .05 in absolute value were significant, before any adjustment for multiple testing. Other MET is energy expenditure when not engaged in formal exercise activities (Total MET less the activity-related METs listed)

**Table 3:** Correlations among Age–Sex Adjusted Study Variables

	1	2	3	4	5	6
(1) Depression Composite	1.000					
(2) Log Depression Composite	.977	1.000				
(3) Cycle Fitness	− .190	− .186	1.000			
(4) Total MET per Day	− .098	− .089	.215	1.000		
(5) Exercise MET per Day	− .119	− .111	.300	.620	1.000	
(6) Other MET per Day	− .074	− .063	.143	.955	.382	1.000

All correlations significant at *p* < .05, with no adjustment for multiple testing. Those greater in absolute value than .07 significant at *p* < .01

The MZ and DZ twin intraclass correlations are shown in Table 4. The indicated heritability of .68 for the Depression Composite was very typical of those from other studies. All the variables indicated genetic influence, but the activity measures to lower degrees than the Depression Composite (.40–.52). Only two variables indicated shared environmental influence: Cycle Fitness and MET for Exercise, Cycle Fitness about .20 and MET for Exercise about .12. Among the various MET measures, MET for Exercise stood out as having patterns of genetic and environmental influences similar to those on Cycle Fitness. We consider first exercise–depression associations because they are the ones most often discussed due to exercise’s potential value in remediating a large public health problem, but then turn to the opposite direction of association. Table 4 also shows the raw variance component estimates and estimated genetic and environmental correlations from the Cholesky models. The variance component estimates were very consistent with the indications from the intraclass twin correlations. As usual, the model estimates were not identical to the twin correlations, but these estimates are usually considered more accurate. For Cycle Fitness and Depression, there was a substantial genetic correlation of − .5 and a non-shared environmental correlation of 1.0. Though MET for Exercise’s variance components were quite similar to Cycle Fitness’, its genetic and environmental correlations differed: −  .06 for genetic, − 1.0 for shared environmental, and − ..13 for non-shared environmental. Other MET also had a shared environmental correlation of − 1.0 with Depression, but the other MET-related correlations with Depression were at most .16 in absolute value.

**Table 4:** Intraclass twin correlations in study variables and Cholesky-indicated variance components and genetic and environmental correlations variable pairs tested for moderation

Intraclass twin correlations	MZ	DZ
Depression Composite	.524	.181
Log Depression Composite	.526	.167
Cycle Fitness	.658	.433
Total MET per Day	.394	.162
Exercise MET per Day	.657	.392
Other MET per Day	.333	.135

Cholesky-indicated variance components and genetic and environmental correlations	Raw A^2^	Raw C^2^	Raw E^2^	rA	rC	rE
Cycle Fitness and Depression	.59	.01	.43	− .50	.00	1.00
Total MET and Depression	.44	.00	.53	− .06	–	− .10
Exercise MET and Depression	.45	.16	.34	− .06	− 1.00	− .13
Other MET and Depression	.21	.03	.50	.16	− 1.00	− .03
Cycle Fitness and Depression	.58	.01	.42			
Total MET and Depression	.46	.00	.54			
Exercise MET and Depression	.47	.17	.36			
Other MET and Depression	.29	.04	.68			

Confidence intervals for genetic correlations ranged .35 to .55 around the estimates. For non-shared environmental correlations, they .14 to .25 around the estimates. Due to small amounts of variance, the shared environmental correlation estimates had no precision

*MZ* monozygotic, *DZ* dizygotic, *A*^*2*^ genetic, *C*^*2*^ shared environmental, *E*^*2*^ non-shared environmental variance, *rA* genetic, *rC* shared environmental, *rE* non-shared environmental correlation

### Exercise–Depression Associations

#### Cycle Fitness Moderating Depression

**Fig. 2:**
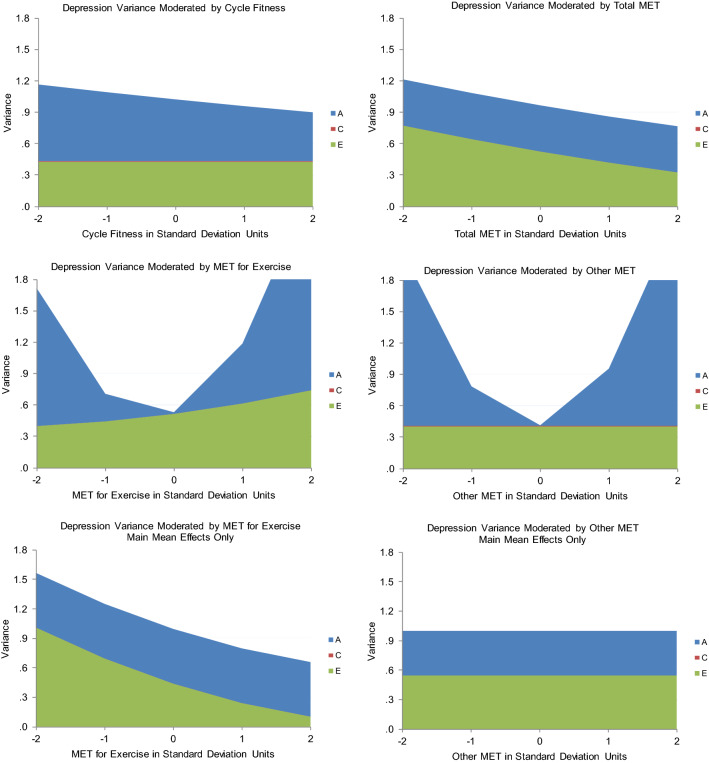
The various exercise-related measures moderating Depression variance from the best-fitting full gene–environment correlation models and the relevant nonlinear models

**Fig. 3:**
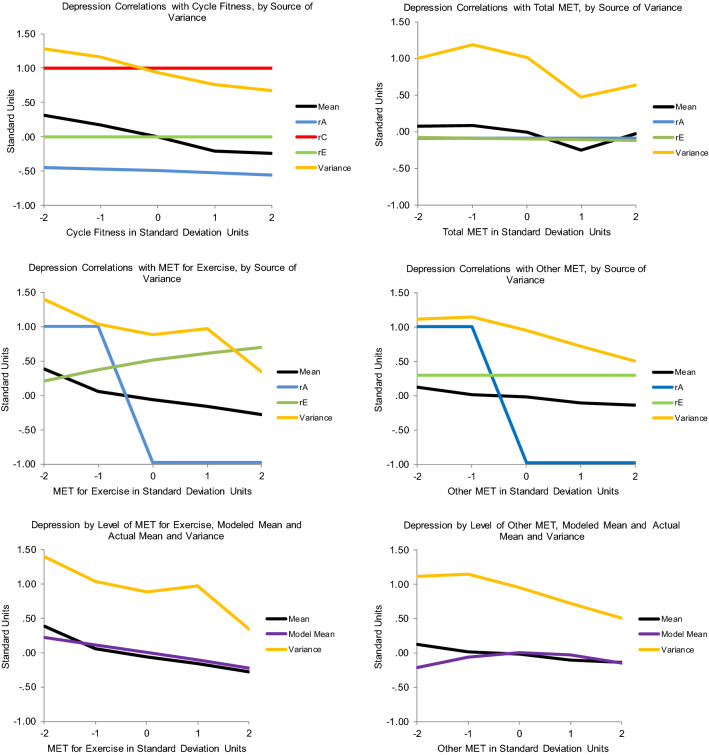
The genetic and environmental correlations from the best-fitting full gene–environment correlation models of the various exercise-related variables moderating Depression and the relevant nonlinear models, along with actual means and total variances by levels of the moderating variables

**Fig. 4:**
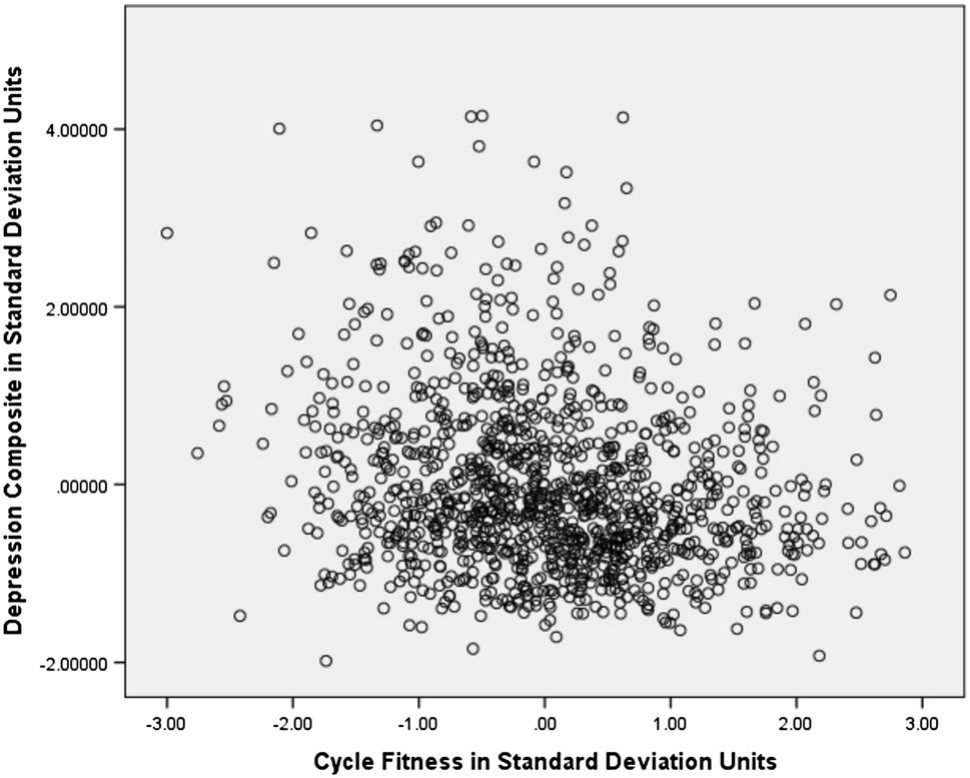
The data to which the Cycle Fitness moderating Depression model was fit. It was similarly possible to see the best-fitting model in each of the others too. Even when the models did not track the general data patterns well, it was possible to see the specific aspects they had captured and exaggerated

As shown in Table 5, the best-fitting model for Cycle Fitness moderating Depression indicated that high fitness suppressed genetic variance unique to Depression. No other variance components were significantly moderated. When Cycle Fitness was 2 standard deviations above the mean, there was only about 2/3s as much genetic variance as when Cycle Fitness was 2 standard deviations below the mean (see Fig. 2, panel 1). There was a ‘scrap’ of shared environmental influence (though the twin correlations indicated more than that), and nonshared environmental influence was stably substantial across the range. The genetic correlation was about −  .5 (see Fig. 3, panel 1), though slightly higher when Cycle Fitness was low, and slightly lower when it was high. This indicated that many genes contributing to good Cycle Fitness also tended to protect against Depression. It was apparently something about Cycle Fitness as an environment that suppressed the genetic influences unique to Depression, primarily by curtailing high levels of expression. The shared environmental correlation was 1.00: whatever environmental factors acted to make all twins similar in Cycle Fitness also similarly protected them from experiencing much depression. This correlation, together with the genetic correlation, probably generated the shared environmental influences suggested by the twin correlations. One way in which this could occur is if Cycle Fitness uniformly reduced Depression somewhat in everyone and genes contributing to tendency towards greater Cycle Fitness also protected against Depression. There was no nonshared environmental correlation, so exercise’s effects seemed to be rooted in factors and habits established over time rather than practices recently initiated. This should probably be expected in a community sample including relatively few with high levels of depression symptoms, and may indicate little about what could or would not be accomplished with exercise in clinical samples. Importantly, the modeled patterns tracked the actual data quite well: crude ‘bins’ 1 standard deviation in length generally indicated less Depression variance at higher levels of Cycle Fitness coupled with lower mean levels as indicated in Fig. 3, panel 1, and see the scatter plot of the data in Fig. 4. They also tracked the Cholesky model estimates well. This indicated that power had been sufficient and the interaction was not a statistical false-positive. There was no evidence that running the ‘Extended Univariate Moderation Model’ suggested by van der Sluis et al. (2012) would be of benefit here.

**Table 5:** Model fit statistics—full gene–environment correlation model

Model	χ^2^ (df)	Δχ^2^ (df, *p*)	AIC	BIC	SSABIC
Cycle Fitness moderating Depression					
All free	3208.68 (1178)	–	852.68	− 1776.40	91.70
Fix all	3222.71 (1184)	14.03 (6, 0.03)	854.71	− 1786.60	91.01
Fix A_C_, C_C_, E_C_, C_U_	3211.47 (1182)	3.03 (4, ns)	847.47	− 1786.48	87.96
*Fix all but A_U_	3211.47 (1183)	3.03 (5, ns)	845.47	− 1789.35	86.68
Fix all but E_U_	3212.26 (1183)	3.58 (5, ns)	846.26	− 1788.96	87.07
Total MET moderating Depression					
All free	5320.87 (1903)	–	1514.87	− 3225.74	− 205.73
Fix all	5331.47 (1909)	10.60 (6, ns)	1513.47	− 3239.00	− 209.47
Fix A_C_, C_C_, E_C_, C_U_	5321.88 (1907)	1.01 (4, ns)	1507.88		− 3237.61
Fix all but A_U_	5324.88 (1908)	4.01 (5, ns)	1508.88	− 3239.20	− 211.26
*Fix all but E_U_	5322.31 (1908)	1.44 (5, ns)	1506.31	− 3240.49	− 212.54
MET for Exercise moderating Depression					
All free	6337.01 (2399)	–	1539.01	− 4781.83	− 972.92
Fix all	6520.22 (2405)	193.21 (6, < 0.001)	1710.22	− 4710.11	− 891.68
Fix C_C_, A_U_, C_U_	6337.01 (2402)	0.00 (3, ns)	1533.01	− 4791.77	− 978.10
Fix all but A_C_	6451.08 (2404)	114.07 (5, < 0.001)	1543.08	− 4797.37	− 974.52
Fix all but E_C_	6413.75 (2404)	76.74 (5, < 0.001)	1705.73	− 4710.04	− 893.20
*Fix all but A_C_, E_C_	6337.45 (2403)	0.44 (4, ns)	1531.45	− 4794.87	− 979.61
Other MET moderating Depression					
All free	5498.58 (2142)	–	1214.58	− 4140.39	− 740.13
Fix all	5701.90 (2148)	203.32 (6, < 0.001)	1405.90	− 4058.93	− 648.25
Fix C_C_, A_U_, C_U_	5498.65 (2145)	0.07 (3, ns)	1208.65	− 4160.00	− 744.98
Fix C_C_, A_U_, C_U_, E_U_	5503.45 (2146)	4.87 (4, ns)	1211.45	− 4150.82	− 744.21
Fix C_C_, E_C_, A_U_, C_U_	5503.65 (2146)	5.00 (4, ns)	1211.65	− 4150.72	− 744.11
*Fix all but A_C_	5504.96 (2147)	6.38 (5, ns)	1210.96	− 4153.28	− 745.09
Depression moderating Other MET					
All free	3057.98 (1199)	–	659.98	− 1898.37	2.95
Fix all	3173.96 (1205)	15.98 (3, 0.003)	763.96	− 1857.93	53.30
Fix C_C_, A_U_, C_U_	3058.79 (1202)	0.81 (3, ns)	654.79	− 1906.54	− 0.47
*Fix all but A_C_, E_C_	3059.87 (1203)	1.89 (4, ns)	653.87	− 1908.06	− 1.20
Fix all but A_C_	3066.00 (1204)	8.02 (5, ns)	658.00	− 1908.65	0.59
Depression moderating MET for Exercise					
All free	3083.76 (1171)	–	741.76	− 1791.80	65.02
Fix all	3201.94 (1177)	18.18 (6, 0.005)	847.89	− 1749.82	116.52
*Fix all but A_U_, E_U_	3085.16 (1175)	1.40 (4, ns)	735.16	− 1802.49	60.68
Fix all but A_U_	3092.13 (1176)	8.37 (5, ns)	740.13	− 1801.85	62.90
Fix all but E_U_	3137.84 (1176)	54.08 (5, ns)	785.84	− 1778.79	85.76
Depression moderating Cycle Fitness					
All free	5644.09 (2052)	–	1540.09	− 3742.91	− 486.64
*Fix all	5649.23 (2058)	5.14 (6, ns)	1533.23	− 3759.54	− 492.74
Fix all but A_U_	5649.21 (2057)	5.12 (5, ns)	1535.21	− 3756.35	− 491.14
Depression moderating Cycle Fitness					
All free	5644.09 (2052)	–	1540.09	− 3742.91	− 486.64
*Fix all	5649.23 (2058)	5.14 (6, ns)	1533.23	− 3759.54	− 492.74
Fix all but A_U_	5649.21 (2057)	5.12 (5, ns)	1535.21	− 3756.35	− 491.14
Fix all but E_U_	5646.30 (2057)	2.21 (5, ns)	1532.30	− 3757.80	− 492.59
Depression moderating Total MET					
All free	6328.00 (2256)	–	1816.00	− 4053.62	− 472.52
*Fix all	6336.51 (2262)	8.51 (6, ns)	1812.51	− 4068.55	− 477.93
Fix all but A_U_	6336.17 (2261)	8.17 (5, ns)	1814.17	− 4065.53	− 476.49
Fix all but E_U_	6334.42 (2261)	6.42 (5, ns)	1812.42	− 4066.40	− 477.37

*A* genetic, *C* shared environmental, *E* nonshared environmental variance, *subscripted C* variance shared between Depression and the exercise variable, *subscripted U* variance unique to the moderated variable, *ns* nonsignificant, *AIC* Akaike Information Criterion, *BIC* Bayesian Information Criterion, *SSABIC* Sample-Size Adjusted Bayesian Information Criterion

*Model judged best-fitting

#### Total MET Moderating Depression

Total MET also suppressed variance unique to Depression, apparently by constraining high levels of expression, but it was the nonshared environmental rather than genetic variance that was suppressed (Table 5; Fig. 2, panel 2). Genetic variance was stably substantial, and there was no shared environmental variance. The genetic and nonshared environmental correlations were effectively 0 (Fig. 3, panel 2). Thus, whatever it was about the environment that distinguished twins in Total MET did not seem to have anything to do with whatever distinguished them in Depression, indicating no direct effects of Other MET on Depression. This model did not track the data quite as well though: it missed that variance in Depression was lowest at 1 standard deviation above mean Cycle Fitness, as was mean level (Fig. 3, panel 2). Total MET confounds general metabolism with physical activity, and the positive full shared environmental correlation between Cycle Fitness and Depression and similarity of the twin correlation patterns between Cycle Fitness and MET for Exercise suggested this confounding may have obscured relevant factors. Again, estimates were quite consistent with those from the Cholesky model, power appeared to have been adequate, and there was no evidence that running van der Sluis et al.’s (2012) suggested model would be of benefit.

#### MET for Exercise Moderating Depression

Results of modeling MET for Exercise moderating Depression, however, were much less clear: the full model tracking both variance common to the two and unique to Depression indicated moderation of both common genetic and common nonshared environmental variance (Table 5), no significant moderation of variance unique to Depression, and no shared environmental variance at all—despite the indications in the twin correlations (Table 4). But these results indicated strong moderation of genetic variance at both extremes of MET for Exercise that did not track the empirical data at all and moderation of nonshared environmental variance was opposite the suppression of total variance at higher levels of depression indicated by the empirical data: compare Figs. 2 and 3, panels 3. They also did not track the Cholesky model estimates. Though the data offered nothing to suggest nonlinear main effects, they strongly suggested confounding of direct effects on level and covariance moderation, so running the nonlinear main effects model was the appropriate next step.

Fit statistics for this model are shown in Table 6. Under it, there was a linear-only main effect of MET for Exercise on Depression of −  .11, and higher MET for Exercise suppressed the nonshared environmental variance in Depression quite sharply, so that at 2 standard deviations above mean MET for Exercise, it was only about 10% what it was at 2 standard deviations below mean MET for Exercise. These results tracked the empirical data and the Cholesky model estimates quite well—see Fig. 2, panel 5 and Table 4. They implied that all twins, but especially MZs, were more similar in Depression at higher levels of MET for Exercise since genetic variance was the same, but total variance lower there. Mean levels of Depression were also lower there, again indicating fewer high scores. The full model hinted at an explanation: its genetic correlation was 1.00 when MET for Exercise was below its mean, but − 1.00 when it was above (Fig. 3, panel 3), which was at least consistent with the very small overall correlation indicated by the Cholesky model. This suggested that genes protecting against depression also tended to encourage exercise, and those contributing to vulnerability also tended to discourage it. The 1.00 shared environmental but negative genetic correlations in the Cycle Fitness model (Fig. 3, panel 1) may have similarly left hints: perhaps performance tends to become important when people engage in high levels of exercise, and if general metabolic factors impede fitness improvement relative to others similarly engaged, this becomes depressing itself. This could act to undermine otherwise beneficial effects of exercise on depression or even fuel depressive symptoms if exercise training has become excessive. This made examining both Other MET’s moderation patterns and those of the other causal direction particularly important, but offered no indication that running van der Sluis et al.’s (2012) would be of benefit.

**Table 6:** Fit statistics for nonlinear models as needed

Model	χ^2^ (df)	Δχ^2^ (df, *p*)	AIC	BIC	SSABIC
MET for Exercise moderating Depression					
All free	2472.28 (895)	–	682.28	− 1631.68	− 210.97
Fix all	2494.94 (899)	22.66 (4, .001)	694.94	− 1633.16	− 206.11
Fix F	2474.33 (896)	2.05 (1, ns)	682.32	− 1633.86	− 211.56
Fix F, A_U_	2474.95 (897)	2.67 (2, ns)	680.95	− 1636.75	− 212.87
Fix F, C_U_	2475.65 (897)	3.37 (2, ns)	681.65	− 1636.40	− 206.11
*Fix all but E_U_	2475.68 (898)	3.40 (3, ns)	679.68	− 1639.59	− 214.12
Other MET moderating Depression					
All free	2555.51 (906)	–	743.51	− 1628.29	− 190.11
*Fix all	2558.06 (910)	2.55 (4, ns)	738.06	− 1639.06	− 195.31
Fix F, C_U_	2557.53 (908)	2.02 (2, ns)	741.53	− 1633.69	− 192.33
Depression moderating Other MET					
All free	1487.88 (599)	–	289.88	− 968.31	− 18.45
*Fix all	1493.32 (603)	6.44 (4, ns)	287.32	− 977.03	− 20.82
Depression moderating MET for Exercise					
All free	1657.28 (599)	–	459.28	− 883.61	66.26
Fix all	1664.04 (603)	6.76 (4, ns)	458.04	− 891.67	64.54
*Fix F, C_U_	1659.78 (601)	2.50 (2, ns)	457.78	− 888.08	64.96
Fix F, C_U_, E_C_	1663.91 (602)	6.50 (3, ns)	459.91	− 888.87	65.75
Fix F, A_U_, C_U_	1663.23 (602)	5.95 (3, ns)	459.23	− 889.21	65.41

*A* genetic, *C* shared environmental, *E* nonshared environmental variance, *subscripted U* variance unique to the moderated variable, *F* quadratic main effect on mean, *ns* nonsignificant, *AIC* Akaike Information Criterion, *BIC* Bayesian Information Criterion, *SSABIC* Sample-Size Adjusted Bayesian Information Criterion

*Model judged best-fitting

#### Other MET moderating Depression

Other MET only significantly moderated genetic variance common to Other MET and Depression (Table 5), but again these results did not track the empirical data or the Cholesky model estimates well at all (compare Figs. 2, 3, panels 4; Table 4). The genetic correlation was again 1.00 when Other MET was below its mean but − 1.00 when Other MET was above its mean (Fig. 3, panel 4), consistent with a generalization of the possibility hinted by the MET for Exercise–Depression correlations: that relatively low metabolic rates in general, whether people are engaged in or aware of their response to physical training or not, also confer vulnerability to depression, and also consistent with the Cholesky model estimates. Though the empirical data did not indicate anything other than a linear main effect, the nonlinear model did (Table 6). Thus the main effect it indicated did not track the data well either (Fig. 3, panel 6): empirically, mean levels of Depression were consistently lower at higher levels of Other MET, but the model indicated they were lowest at the two extremes of Other MET. And the data indicated that total Depression variance was suppressed at high levels of Other MET, but the nonlinear model indicated no moderation—at least of the variance unique to Depression. Neither of the models applied handle situations in which direct uniform main effects are confounded with moderated covariance well, and power to address shared environmental variance is always lowest in quantitative genetic models. The shape of the indicated nonlinear main effect in that model may have been hinting at the same confounded factors as the 1.00 shared environmental correlation in the Cycle Fitness model and the poor tracking of the data in the full moderation models of Total MET and MET for Exercise, coupled with the latters’ − 1.0 genetic correlations below mean MET levels and 1.0 genetic correlations above them: something about low Other MET’s genetic association with depression vulnerability undermined otherwise beneficial nonshared environmental effects of exercise on Depression, perhaps especially when it impeded physical training response in people interested in performance, and maybe particularly so when this was apparent relative to close others (such as co-twins—which would manifest as nonshared environmental influence). As with the other Depression moderators, the data did not suggest that running van der Sluis et al.’s (2012) model would be of benefit.

### Depression–Exercise Associations

#### Depression Aoderating Other MET

**Fig. 5:**
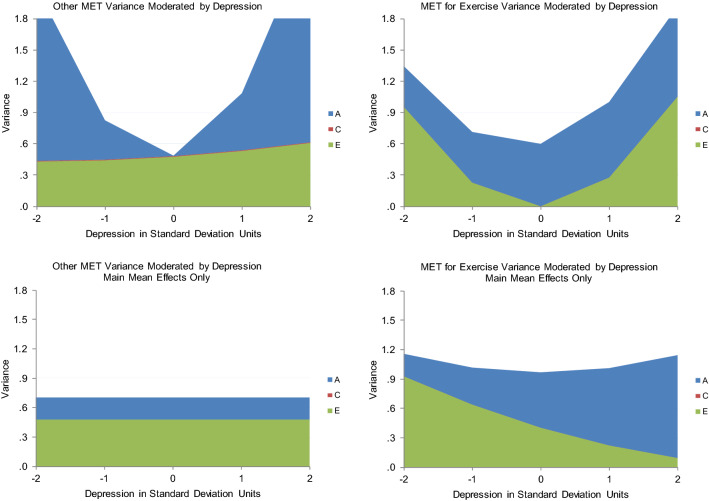
Depression moderating the various exercise-related measures’ variances, from the best-fitting full gene–environment correlation models and the relevant nonlinear models in which moderating effects and/or nonlinear main effects were significant

**Fig. 6:**
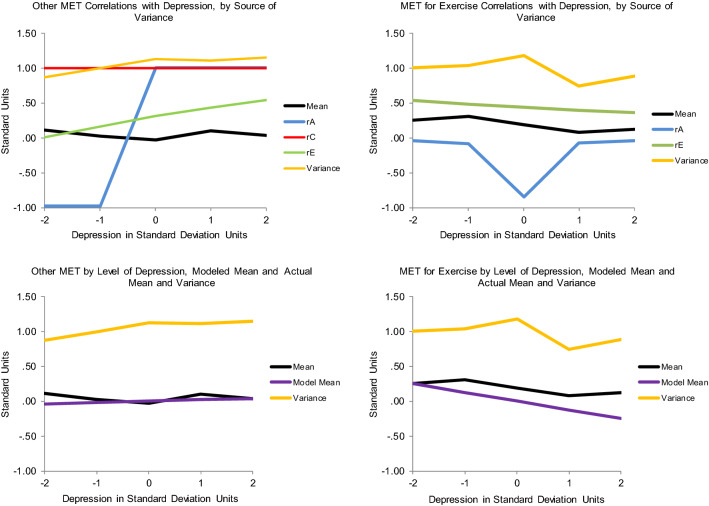
The genetic and environmental correlations from the best-fitting full gene–environment correlation models of Depression moderating the relevant exercise-related variables and nonlinear models in which moderating effects and/or nonlinear main effects were significant, along with actual means and total variance by levels of Depression

Given the general possibility raised by the model-indicated exercise–depression associations that low metabolic rate and vulnerability to depression may be inter-related, Depression moderating Other MET seemed the place to start examining this causal direction. Depression moderated both genetic and nonshared environmental influences common to the two (Table 5), but again the indications did not track the data at all well (compare Figs. 5, 6, panels 1). Consistent with the other direction of association, the genetic correlation took opposite signs below and above mean Depression, but the signs were reversed: the correlation was − 1.0 when Depression was below its mean and 1.0 when it was above (Fig. 6, panel 1). There was a little shared environmental covariance but no shared environmental variance unique to Other MET, generating a 1.0 shared environmental correlation. The actual data indicated somewhat more variance at high levels of Other MET, but the nonlinear model indicated no significant moderating effects and no nonlinear main effects (Table 5; Fig. 5, panel 3), and mean levels of Other MET were effectively flat across levels of Depression. This suggested that, in the association between Other MET and Depression, Other MET was the primary driver. Effects of depressive symptoms on getting out and exercising were more likely, however, so we examined whether Depression moderated MET for Exercise variance next.

#### Depression Moderating MET for Exercise

The full moderation model indicated genetic and nonshared environmental moderation of variance unique to MET for Exercise (Table 6; Fig. 5, panel 2), with genetic correlations of 0 when Depression was either low or high and − 1.0 in the mid-ranges (Fig. 6, panel 2). But it did not track the actual data well at all: it indicated great nonshared environmental variance in MET for Exercise at both low and high levels of Depression and none in the mid-range, while the actual data indicated perhaps an overall suppression, but with *more* variance in the mid-ranges of Depression (compare Figs. 5, 6, panels 2). We therefore fit the nonlinear model too. It also indicated moderation of genetic and nonshared environmental variance (Fig. 5, panel 4), but genetic variance unique to MET for Exercise was suppressed when Depression was *low* and nonshared environmental variance when it was *high* (Fig. 6, panel 4)*.* This pattern was far less dramatic, but not accurate either, and the mean-level indication also did not track the data well. It appeared that, whatever else might have gone on, the primary driver in the association between Depression and MET for Exercise was MET for Exercise. For completeness and to emphasize the point, neither model indicated any moderating or nonlinear main effects of Depression on either Cycle Fitness or Total MET.

None of these models offered any indication that running van der Sluis et al.’s (2012) model would be of benefit either.

## General Discussion

We hypothesized that exercise would constrain variance in expression of depression symptoms, and particularly their genetic variance, tested this, and further explored the various genetic and environmental pathways involved in a community-based twin sample well representing the relatively healthy adult population of Denmark that had completed an unusually extensive set of relevant measures. Our major hypothesis was largely supported: genetic variance in depressive symptoms was suppressed at high levels of the two most directly activity based measures, Cycle Fitness and MET for Exercise. Moreover, the genetic variance suppression was reinforced for MET for Exercise by suppressed nonshared environmental variance. But there were strong indications throughout our analyses that neither exercise measure was free of influence from general body metabolism, and that genetic influences on low body metabolic rate appeared also to confer vulnerability to depression symptoms.

We also considered the possibility that depressive symptoms such as low motivation to do much of anything or fatigue from lack of sleep might suppress expression of otherwise-existing proclivities to exercise, but this did not appear to be the case to any noticeable degree. This may to some extent have been specific to this sample and its particular measures of depression. There were probably very few cases of clinical depression and the NEO scale and possibly also the other scales may primarily reflect chronic depression symptoms rather than state depression itself. For example, lack of energy and fatigue are among the criteria for clinical depression in ICD-10, and these can arise and be chronic for many reasons besides depression.

Two pathways appeared likely involved in suppressing variance in depressive symptoms at high levels of exercise. The first related to general metabolic function and probably involved mostly genetic variance: exercise might constrain expression of genes contributing both to keeping general metabolic rate low and depressive symptoms. The second could be a source of the apparent genetic and environmental and measure-related general metabolic confounds we observed: people who ‘get into’ exercising may also tend to ‘get into’ performance in the forms of exercise in which they are involved (whether formally competing or not) and genetic influences on low general metabolic rate may be among the factors that contribute to the commonly observed tendency of some people to show relatively little fitness response and/or only relatively slowly to physical training. This may be confer an independent genetic and/or environmental vulnerability to depression symptoms, and perhaps especially when close others similarly involved (such as co-twins) are showing greater and/or more rapid response.

Confirmation of these possibilities from other studies could explain the inconsistent results from studies of exercise effects on depressive symptoms in the literature. Our observations could be tested and extended using data from activity-tracking watches that continuously monitor heart rate, and activity data (steps, exercise sessions) could be combined with depression symptom experience sampling to examine exercise–depression associations in more depth. Exercise may ‘work’ rather generally to remediate depressive symptoms, but when people start to care about performance too and seeing improvement seems difficult, this could be especially discouraging to people already rather depressed and undermine any depression remediating effects they might otherwise get from the exercise. This suggests that exercise interventions should be careful, especially in the early intervention stages, of comparing participants’ improvements with each other. They could also actively discourage between-person comparisons by focusing closely on enjoyment of the activity for its own sake and, to whatever extent improvement in performance is noted, tracking increases in enjoyment too and only within individuals. Ways to accomplish these goals successfully should, however, be tested for effectiveness in accomplishing these goals before being ‘writ large’.

### Study Limitations and Strengths

Unfortunately, all too often study strengths bring with them practical limitations, and this one was no exception. Though its measures of involvement in exercise were more extensive than in many studies and included objective assessment (the Cycle Fitness test) and some ability to distinguish general metabolic energy expenditure from that resulting from intended exercise (the MET measures), the latter ability was crude and the former restricted the sample to those healthy enough to undergo its physically stressful nature. Extensive measures require greater investment of time and resources by both researchers and participants, inevitably limiting participant numbers and thus power, and our sample was only of moderate size. Still, it was drawn from a much larger sample highly representative of its population, and was quite representative of that sample. But like most, the population it represented hailed from one country, Denmark in this case, at one time in its history, limiting generalizations to others. Relative to some other economically ‘developed’ countries such as the United States, the Danish population is both more likely to cycle or walk for transportation (and exercise, economy, and environmental conservation reasons), and tends to have lower levels of depression. Relatively low levels of depression were likely especially true of our sample, due to the physical health restrictions imposed for the Cycle Fitness test. This likely limited the relevance of some of our observations to clinical samples somewhat, but depression tends to manifest dimensionally rather than categorically and people tolerate different levels of distress before seeking clinical help even when it is readily available, making any distinction between community and clinical samples arbitrary.

Moreover, equipment failure during testing rendered 268 participants’ Cycle Fitness test results unusable, in the process restricting our ability to estimate Total MET. Because the cause of missingness was unrelated to scores, however, these data could be considered missing at random, so our parameter estimates were unbiased given our use of maximum likelihood estimation (Little and Rubin 1987). Still, the missingness reduced the precision of our parameter estimates. Our MET for Exercise and Depression measures were self-report and thus subject to the usual limitations of such measures, including memory failure, reporting biases, and, for the measures of intensity, individual differences in perception of intensity of activity. They were, however, quite extensive, in particular the assessment of physical activity, which separately addressed frequency of involvement and intensity of activity during work, for transportation, and at leisure. Still, participants could have included their activities in more than one of our activity categories, for example cycling both for transportation to work and on errands and during leisure time for recreation or explicit training and including total cycling time in both categories, resulting in duplicated reports. In addition, we based our estimates of METs for these activities obtained from overall averages from studies based usually on American samples that may not represent our sample well, and certainly did not apply equally well to all participants, as energy expenditures during activity and metabolic rates show large individual differences.

Perhaps mostly importantly, this study, not for the first time, revealed clearly the limitations of currently available methods to assess gene–environment interplay, especially their correlation. Though we were careful to avoid false positives due to distributional properties, as noted by Eaves (2017) recently and Falconer and McKay back in 1989, our models’ inability to handle apparently moderated covariance between moderator and outcome variables, which we have noted many times and van der Sluis et al. (2012) also pointed out, was clearly apparent in our results. The field simply needs much better models to identify and characterize manner and sources of gene–environment correlation, in general but especially when, as is often (Johnson 2007), it presents along with gene–environment interaction.

### Conclusions and Recommendations for Future Study

We tested whether exercise suppressed variance in depression symptoms and examined the genetic and environmental pathways involved to further understanding of the reasons for inconsistency of study findings regarding exercise as a remedy for depression and the processes involved when it appears to be effective. We generally observed the hypothesized suppression of genetic variance, but also confounding environmental and measure-related factors that appear to implicate more complex gene–environment interplay that may imply different effects in different populations in different circumstances rather generally. These factors should receive further test and application of better methods badly in need of development. But if confirmed, they suggest care in developing exercise interventions as treatments for depression, so that they avoid inter-individual comparisons and competition and focus on developing enjoyment of exercise for its own sake over thinking about quality of performance.

## Footnotes

1 Factor and principal components analyses of the 3 variables very clearly indicated a single factor or component, and the variables’ loadings were high and very similar, ranging .76–.83 under factor analysis and .85–.88 under principal components analysis. As we had no conceptual reason to consider any of the variables more or less valid indicators of depression so weighting them by factor or component loadings would be solely a statistical manipulation, we opted to weight them equally as a composite variable by averaging them in standardized form. This decision had effectively no impact on our results, as the correlation between the composite and factor-score variables was .998. Their distributional properties were highly similar as well.
